# Equipment-related wounds and associated risk factors in working equids of the Oromia national regional state in Ethiopia

**DOI:** 10.1017/awf.2024.52

**Published:** 2024-10-31

**Authors:** Mathilde S Merridale-Punter, Abel L Wodajo, Belay Elias, Anna-Marie Bakos, Hanna Zewdu, Reta Tesfaye, Gizachew Hailegebreal, Teshale Sori, Charles M El-Hage, Anke K Wiethoelter, Peta L Hitchens

**Affiliations:** 1 Melbourne Veterinary School, Faculty of Science, University of Melbourne, Parkville, VIC 3010, Australia; 2 College of Veterinary Medicine and Agriculture, Addis Ababa University, PO Box 34, Bishoftu, Ethiopia; 3 Faculty of Veterinary Medicine, Hawassa University, PO Box 05, Hawassa, Ethiopia; 4 Equine Centre, Melbourne Veterinary School, University of Melbourne, 250 Princes Hwy, Werribee, VIC 3030, Australia

**Keywords:** animal welfare, cross-sectional, donkeys, equipment-related wounds, harness, horses

## Abstract

Working equids support the livelihoods of millions of low-income households worldwide and face several welfare challenges. Although equipment-related wounds are common, little is known about specific risk factors. This cross-sectional study surveyed equids used for cart-work in three Ethiopian towns. Number, size, severity and location of wounds were recorded for each animal, as well as work equipment characteristics and indicators of equipment fit and assembly. Questionnaires were conducted with each cart-driver focusing on equipment practices and attitudes. Logistic regression models were generated to investigate associations between equipment-related wounds (outcome) and equipment, work, driver and animal-related factors (predictors). In total, 369 equids and cart-drivers were surveyed. The prevalence of equipment-related wounds was 72.6% (268/369) with girth wounds being the predominant wound type in horses (50%; 122/244) while donkeys had predominantly shaft-related wounds (59%; 72/122). Donkeys were two times more likely to have equipment-related wounds than horses. The presence of equipment-related wounds was associated with factors such as previously having wounds, inadequate collar positioning and using purchased equipment compared to partly home-made equipment. Characteristics of specific equipment components were also associated with certain wound types, such as narrow saddle pressure points and saddle wounds. Equipment-related wounds are highly prevalent in working equids, representing a serious welfare concern. Factors relating to equipment design, fit and assembly were associated with the presence of wounds. Importantly, species differences require tailoring of preventive approaches amongst working equids. A better understanding of work equipment should therefore be promoted as part of wound prevention and animal welfare strategies.

## Introduction

Working equids support millions of low- and middle-income households worldwide, often being a central source of income and playing a key role in the wider community through their use in transport, agriculture, domestic support and supply chains (Fernando & Starkey 2004; Sturgeon 2021; Grace *et al.* 2022). Of all the African nations, Ethiopia has the highest number of equids (Food and Agriculture Organisation [FAO] 2021; Norris *et al.* 2021), where over 12 million are estimated to be used for transport and other draught or pack work (Central Statistical Agency [CSA] 2020; Geiger *et al.* 2020).

With working equids frequently working every day of the week (Bazezew *et al.* 2014; Ali *et al.* 2016), work equipment is an essential part of their lives, and can impact their welfare (Ali *et al.* 2016; Teferi *et al.* 2020; Eriso *et al.* 2023a). Equipment-related wounds are one of the most visible manifestations of welfare compromise attributable to equipment and are widely prevalent in working equids around the world (Farhat *et al.* 2020; Robledo-Reyes *et al.* 2020; Rodrigues *et al.* 2020). In Ethiopia, wounds in the region of the harness have been commonly reported (Teferi *et al.* 2020; Aliye *et al.* 2022), with prevalence varying from 13% (Eriso *et al.* 2023b) to as high as 60% (Fikru *et al.* 2015) or 80% (Usman *et al.* 2015) in different regions. Further, the presence of wounds has also been linked to other conditions such as lameness (Ali *et al.* 2016), suggesting that inadequate harnessing can not only impact the animal directly but also carries broader indirect welfare implications.

Work equipment characteristics such as harness materials and fitting are described in harnessing guidelines as a potential risk for injury (Pearson *et al.* 2003; Heleski *et al.* 2009; Garrett 2017), and the type of work and harness design have been shown to affect the presence of wounds (Daneil & Asmare 2013; Ali *et al.* 2016). Some studies have reported harness cleanliness (Farhat *et al.* 2020), loads carried (Fikru *et al.* 2015; Ali *et al.* 2016), and poor general harness quality causing rubbing or friction to be associated with wounds (Ali *et al.* 2016; Bereket & Addis 2019). However, previous surveys have classified harnessing equipment broadly as appropriate or inappropriate, with few studies examining the specific equipment attributes that represent risk factors for the development of wounds. Equid harnesses and carts are complex work equipment with multiple interacting components that act as a unit and depend upon other elements to be adequately functional, efficient and comfortable (Pearson *et al.* 2003; Oudman 2004). Additionally, the way in which this equipment fits the animal and is hitched, as well as the way in which it is used and kept by the driver likely impacts the risk of developing equipment-related wounds (Pearson *et al.* 2003; Garrett 2017; Rodrigues *et al.* 2022).

Guidelines are already available for affordable, efficient and welfare-friendly equipment designs (Pearson *et al.* 2003; Heleski *et al.* 2009; Garrett 2017) but implementation of such guidelines remains challenging with some reports suggesting targeted improvement and modification of existing work equipment may be more successful in effecting change than radical modifications or introduction of new equipment designs (Chanie *et al.* 2012). Therefore, a better understanding of the specific equipment attributes and practices associated with the presence of equipment-related wounds would provide valuable insight into preventing this welfare risk to working equids.

This study therefore aimed to: (1) determine the prevalence of equipment-related wounds in working equids at three Ethiopian locations; and (2) investigate the association between equipment-related wounds (outcome) and work practices, cart-driver demographics and animal and equipment characteristics (predictors). A greater understanding of which work equipment and driver characteristics or practices are associated with the presence of wounds will help devise recommendations that may be implemented by cart and harness makers, as well as informed wound-prevention strategies for working equid owners and users.

## Materials and methods

### Study design and data collection

Full methodology for data collection and sample size calculation are described in detail in Merridale-Punter *et al.* (2024). In brief, this was a cross-sectional study conducted in three locations in the Oromia national regional state of Ethiopia, within the East and North Shewa zones (Bishoftu and Fiche towns, respectively) as well as the West Arsi Zone (Shashamene town). Data were collected between February and April 2022. The study population consisted of working equids (defined in this study as donkeys, horses or mules used for work that involves wearing a harness hitched to a cart) and their corresponding cart-drivers. Recruitment of participants took place via random systematic sampling frame and occurred at dedicated cart-holding stations selected on the basis of convenience within each of the study regions.

A sample size of 369 working equids and their drivers was determined, based on a 20% expected prevalence of equipment-related wounds in the study regions, estimated from previous reports of 17% prevalence in Shashamene (Teferi *et al.* 2020) and 18% in Bishoftu (Chala *et al.* 2017). Desired absolute precision was set at 5% and adjustments for clustering variations using a 1.5 design effect inflation (Stevenson 2015). Data collection sheets used for the survey of animal welfare and work equipment indicators, as well as a structured questionnaire with the corresponding cart-drivers are presented in Supplement 1 in the Supplementary material. For each animal, signalment, work type and work intensity indicators were recorded. Animal welfare indicators were selected from and collected in accordance with the validated Standardised Equid-Based Welfare Assessment Tool (SEBWAT) (Sommerville *et al.* 2018). The number, size and severity of wounds (Sommerville *et al.* 2018) were recorded for each animal based on wound location. For each surveyed animal, selected cart and harness characteristics, their fit, materials, design and presence of standard equipment components (Pearson *et al.* 2003; Oudman 2004; Davis 2014; Garrett 2017) were recorded (Merridale-Punter *et al.* 2024). Data collection was carried out by two assessors formally trained for the purpose (ALW and BE).

### Ethical approval

The study received ethical approval from the Human and Animal Research Ethics Committees of the University of Melbourne (Reference ID: 2021-22261-24285-4 and 2021-22886-21887-1, respectively), and from the Animal Research Ethics Review Committee of the Addis Ababa University (Reference: VM/ERC/36-37/07/13/2021). Each cart-driver provided informed consent to participate in the study and was compensated for their time with a pre-paid bag of animal feed.

### Data processing and analysis

Data collected were entered into REDCap (Harris *et al.* 2009, 2019), a secured electronic data capture software hosted at Melbourne Veterinary School. R software (R Core Team 2021) was used to analyse the data descriptively and perform inferential statistical analysis. All equid surveys were complete and included in the study (n = 369); one cart-driver questionnaire was incomplete and removed from analysis (n = 368). Wounds located in the region of the bit, blinkers, chest-band, girth, saddle and shafts, as well as limb and tail wounds in contact with equipment were classified as ‘equipment-related wounds’. Wounds located in other body parts were classified as ‘other wounds’. Work type was recorded as the approximate percentage of time the equid spent performing each given type of work. A categorical variable ‘main type of work’ was then created to reflect the predominant work type, where ‘taxi’, ‘transport of goods’ and ‘transport of water’ were considered when the percentage of time performing this work was higher than any other work types, and with ‘mixed work’ referring to equids performing different types of work in similar amounts. Additional variables were created to reflect the functionality of interacting harness components, as well as to classify harness contact surface materials as ‘breathable’ or not according to whether they were made from natural fibres or synthetic, non-breathable materials, respectively (Heleski *et al.* 2009; Merridale-Punter *et al.* 2024).

Associations between the presence of equipment-related wounds as a binary categorical variable (outcome) and individual equipment characteristics, work practices, species, location, driver demographic and attitudes (predictors) were tested using univariable logistic regression models (see Supplement 2 in the Supplementary material). Due to the low number of mules surveyed (n = 3), only horses and donkeys were considered when investigating associations between species and equipment-related wounds. Collinearity between predictors were tested by pair-wise correlations and Chi-squared tests for associations between categorical variables. Predictor variables from the univariable logistic models with a significance level of *P* < 0.25 were retained and used to generate a directed acyclic graph (DAG) using DAGitty (available on www.dagitty.net; Textor *et al.* 2016) to investigate potential causal pathways between predictors and equipment-related wounds. To generate the DAG, retained predictors were grouped into categories of shared qualities and the predictor that had the highest significance level or was the best biological representation of each category was included in the analysis. Multivariable logistic regression models were then built to estimate the total effect of each predictor category on equipment-related wounds, with minimal sufficient covariate adjustments for the other predictors in the DAG.

Further univariable logistic regression models were used to investigate specific predictors of individual equipment wound types as the outcome (saddle, girth, chest-band, bit, blinker and shaft wounds, and limb and tail wounds attributable to equipment) (see Supplement 2; Supplementary material). Regression coefficients (coef), Odds ratios (OR) and 95% confidence intervals (95% CI) are reported. Statistical significance was set at *P* < 0.05.

## Results

### Animal and equipment survey

Three-hundred and sixty-nine working equids were surveyed between the three study locations, of which 66.1% (244/369) were horses, 33.1% (122/369) were donkeys and 0.8% (3/369) were mules. All equids were male (100%; 369/369) with an overall mean (± SD) body condition score (BCS) out of 5 of 2 (± 0.6). For 63.1% (233/369) of equids, taxi work was the main type of work performed, whereas 28.7% (106/369) spent most of their work time transporting goods, doing mixed work (5.1%; 19/369) or transporting water (3.0%; 11/369). Animals worked a median of six days per week (inter-quartile range [IQR] 6-6 days) and 10 h of work per day (IQR 8–10 h). The median number of breaks taken during the workday was two (IQR 1–2 breaks) lasting a median of 60 min per break (IQR 30–60 min). Three percent of drivers (11/368) only removed the harness during breaks sometimes and 4.9% (18/368) did not remove it. Welfare parameters by species and location are summarised in Supplement 3 in the Supplementary material.

**Figure 1.**
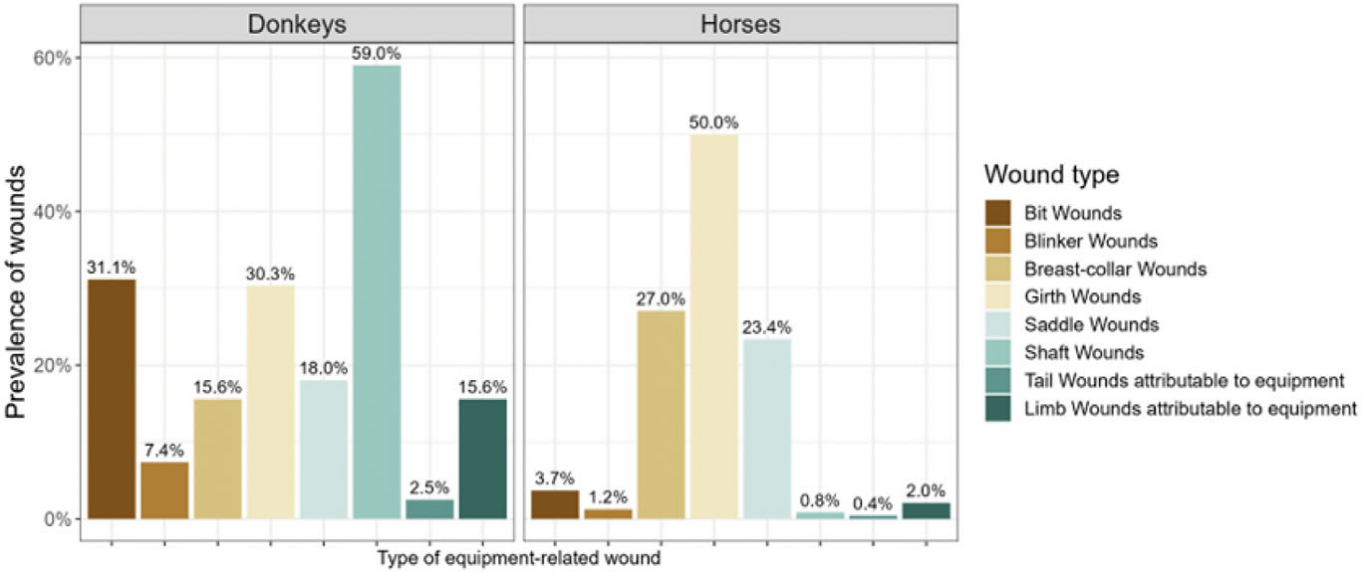
Overall prevalence of different types of equipment-related wounds in a cross-sectional study investigating equipment-related wounds and associated factors in working equids at three Ethiopian locations in 2022.

The overall prevalence of any type of wound was 75.9% (280/369; 95% CI 71.1–80.1%), with a prevalence of equipment-related wounds of 72.6% (268/369; 95% CI 67.7–77.1%). Eighty-two percent of donkeys (82%; 100/122) and 67.6% (165/244) of horses had equipment-related wounds. Overall, girth wounds were the most prevalent type of equipment-related wound among surveyed equids (43.9%; 162/369), followed by breast collar (23.3%; 86/369) wounds, saddle (21.7%; 80/369), shaft (20.3%; 75/369), bit (12.7%; 47/369), limb (6.5%; 24/369), blinker (3.3%; 12/369) and tail wounds (1.1%; 4/369). When stratifying by species, girth wounds (50%; 122/244) were the most common wound type in horses, while in donkeys shaft (59%; 72/122) and bit (31.1%; 38/122) wounds were the most prevalent (Figure 1). Median scores and IQRs for the size, severity and number of each wound type are summarised in Supplement 3 (Supplementary material).

### Cart-driver questionnaires

Findings of the cart-driver questionnaires are detailed in Merridale-Punter *et al.* (2024). Briefly, four drivers were female (1.1%; 4/368) and median cart-driver age was 28 years (IQR 22–35 years). The median cart-driving experience was four years (IQR 3–6 years), and 6.8% of respondents (25/368) were exclusively cart-drivers while 93.2% (343/368) both owned and drove the equid. The majority of respondents purchased their work equipment (84.7%; 310/366), while some (8.5%; 31/366) reported partly purchasing and partly home-making it. A total of 76.6% (282/368) of cart-drivers reported their equid had a previous history of wounds, and 28.8% (106/368) actively took measures to prevent wounds. The most common measure to prevent wounds was taking the animal to a veterinary professional (42.5%; 45/106) (Table 1). Questionnaire responses are described in Supplement 4 in the Supplementary material.

**Table 1.** Equid wound history and wound prevention approaches of cart-drivers in a cross-sectional study investigating equipment-related wounds in Ethiopian working equids in 2022. Overall equids include horses, donkeys and mules, although due to the low frequency of mules (n = 3) species comparison is presented for horses and donkeys only

	Horses		Donkeys		Overall equids	
	n = 243	%	n = 122	%	n = 368	%
Previous history of wounds	171	70.4%	109	89.3%	282	76.6%
Frequency of wound history:						
*Rarely*	68	28%	43	35.2%	112	30.4%
*Occasionally*	84	34.6%	25	20.5%	109	29.6%
*Often*	18	7.4%	37	30.3%	55	14.9%
*Very Often*	1	0.4%	4	3.3%	6	1.6%
Took measures to prevent wounds	80	32.9%	25	20.5%	106	28.8%
Wound prevention measures:						
*Seek veterinary care*	28	11.5%	17	13.9%	45	12.2%
*Wash or cover developing wounds*	27	11.1%	7	5.7%	34	9.2%
*Changing the equipment*	17	7%	1	0.8%	19	5.2%
*Traditional treatments*	8	3.3%	0	0%	8	2.2%

### Factors associated with equipment-related wounds

#### Overall presence of equipment-related wounds

Table 2 presents significant predictors of equipment-related wounds found on univariable analysis.

**Table 2.** Univariably significant predictors of equipment-related wounds in a study investigating the prevalence and factors associated with equipment-related wounds in Ethiopian working equids in 2022. Comparison of species was done between horses (n = 244) and donkeys (n = 122) only

Significant predictor of overall equipment-related wounds	Wounds	No wounds	Odds Ratio (OR)	95% Confidence Interval (OR)	Significance value (*P*)
**Town**					
Shashamene	83.1% (103/124)	16.9% (21/124)	1.00		
Bishoftu	68.9% (84/122)	31.1% (38/122)	0.45	0.25–0.83	0.010
Fiche	65.9% (81/123)	34.1% (42/123)	0.39	0.22–0.72	0.002
**Species**					
Horse	67.6% (165/244)	32.4% (79/244)	1.00		
Donkey	82% (100/122)	18% (22/122)	2.18	1.28–4	0.004
**Main type of work**					
Taxi	66.5% (155/233)	33.5% (78/233)	1.00		
Transport of goods	81.1% (86/106)	18.9% (20/106)	2.16	1.24–3.78	0.007
Mixed work	94.7% (18/19)	5.3% (1/19)	9.06	1.19–69.10	0.034
**Traction through point of draught (POD)**					
Through POD	64.4% (85/132)	35.6% (47/132)	1.00		
Not through POD	77.1% (182/236)	22.9% (54/236)	1.86	1.17–2.98	0.009
**Presence of traces**					
Traces present	67.5% (166/246)	32.5% (80/246)	1.00		
Traces not present	82.8% (101/122)	17.2% (21/122)	2.32	1.35–3.98	0.002
**Presence of neck-strap**					
Neck-strap present	64.9% (137/211)	35.1% (74/211)	1.00		
Neck-strap not present	82.8% (130/157)	17.2% (27/157)	2.60	1.57–4.30	< 0.001
**Presence of swingle tree**					
Swingle tree present	66.9% (101/151)	33.1% (50/151)	1.00		
No swingle tree	76.5% (166/217)	23.5% (51/217)	1.61	1.02–2.56	0.043
**Source of equipment**					
Partly purchased & home-made	51.6% (16/31)	48.4% (15/31)	1.00		
Purchased	74.2% (230/310)	25.8% (80/310)	2.70	1.27–5.70	0.010
Inherited	91.7% (11/12)	8.3% (1/12)	10.31	1.18–89.79	0.035
**Cost of cart** *(per 100 ETB unit; Coef.)*	*n/a*	*n/a*	–0.04	–0.07– –0.01	0.004
**Wound history**					
No history of wounds	34.9% (30/86)	65.1% (56/86)	1.00		
History of wounds	84% (237/282)	16% (45/282)	9.83	5.69–17	< 0.001
**Measures to prevent wounds**					
Taking measures	80.2% (85/106)	19.8% (21/106)	1.00		
Not taking measures	69.5% (182/262)	30.5% (80/262)	0.56	0.33–0.97	0.038

Collinearity was present between several predictor variables. Town and species were strongly associated, where the surveyed population in Fiche and Bishoftu was made up almost entirely of horses (98.8%; 242/245) while equids surveyed in Shashamene were almost exclusively donkeys (96.8%; 120/124; χ^2^ = 348.3; *P* < 0.001).

**Figure 2.**
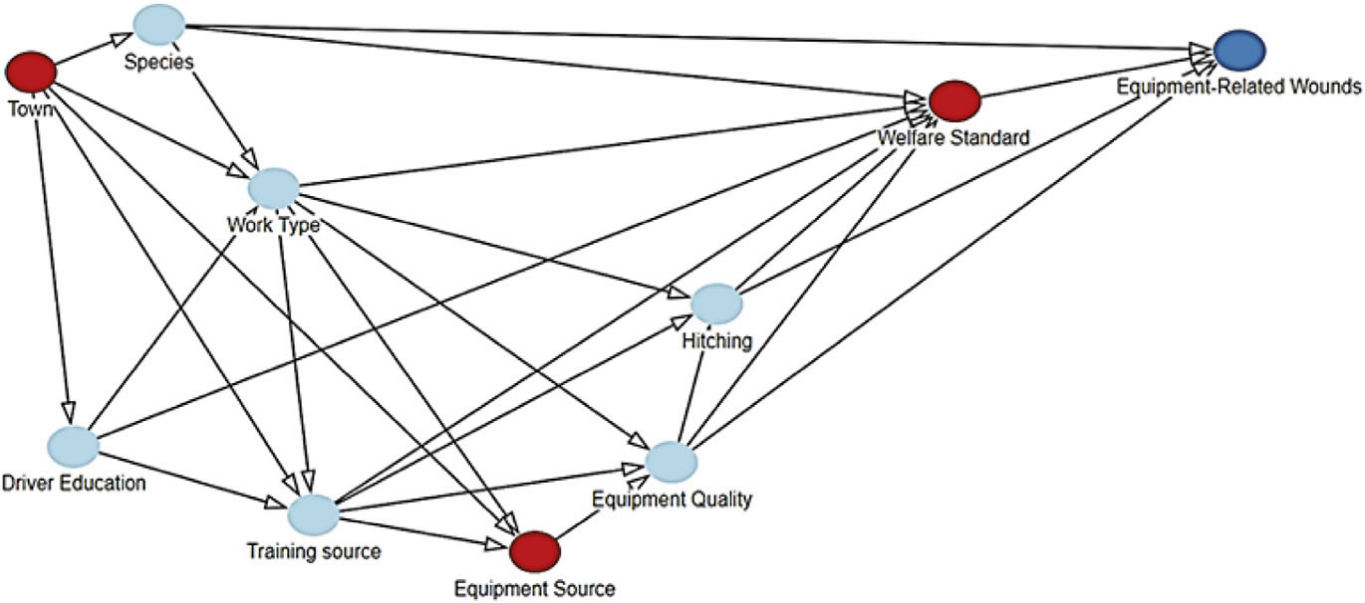
Directed acyclic graph (DAG) representing possible causal pathways between predictors of equipment-related wounds, in a cross-sectional study investigating prevalence and factors associated with equipment-related wounds in Ethiopian working equids in 2022. Only predictors with a significance level of *P* < 0.25 in univariable logistic regression analysis were retained and represented in the DAG. Collinearity between predictors was present and town was associated with all other predictors in the DAG. Predictors coloured in red represent significant predictors of equipment-related wounds after adjustments (*P* < 0.05) and those in light blue represent non-significant variables. The model outcome (equipment-related wounds) is represented in darker blue.

Nine predictor categories were created in the DAG based on retained predictors with a significance level of *P* < 0.25: ‘town’, ‘species’, ‘main work type’, ‘driver education’, ‘source of equipment’, ‘training source’ (including source of training and source of information on hitching and assembly; represented by training source), ‘equipment quality’ (including cost of cart, presence of traces, neck-strap, swingle, breeching, and equipment receiving maintenance; represented by cost of cart), ‘hitching’ (including collar traction through point of draught and presence of functional breeching; represented through traction through point of draught) and ‘welfare standard’ (including BCS, history of wounds and measures to prevent wounds; represented by history of wounds) (Table 3; Figure 2).

**Table 3.** Results of the directed acyclic graph (DAG) -based statistical modelling of the total effects of exposure variables on the likelihood of equipment-related wounds, in a cross-sectional study investigating prevalence and factors associated with equipment-related wounds in Ethiopian working equids in 2022. Exposure variables within DAG category were *P* < 0.25 in univariable analysis

Grouped category represented in the DAG	Variables within DAG category	Minimum sufficient adjustments for estimation of total effect of exposure on equipment wounds	Variable representing DAG category in the model	Equipment related wounds	No equipment related wounds	Odds Ratio (OR)	95% Confidence Interval	Significance value (*P*)
**Town**	**Town**	No adjustments	**Town**					
			Shashamene	83.1% (103/124)	16.9% (21/124)	1.00		
			Bishoftu	68.9% (84/122)	31.1% (38/122)	0.45	0.25–0.83	0.010
			Fiche	65.9% (81/123)	34.1% (42/123)	0.39	0.22–0.72	0.002
**Species**	**Species**	Town	**Species**					
			Horse	67.6% (165/244)	32.4% (79/244)	1.00		
			Donkey	82% (100/122)	18% (22/122)	0.34	0.03–4.21	0.400
**Main type of work**	**Main type of work**	Driver Education, Species, Town	**Main type of work**					
			Taxi	66.5% (155/233)	33.5% (78/233)	1.00		
			Transport of Goods	81.1% (86/106)	18.9% (20/106)	1.37	0.27–6.9	0.699
			Transport of Water	81.8% (9/11)	18.2% (2/11)	1.08	0.11–10.41	0.949
			Mixed work	94.7% (18/19)	5.3% (1/19)	5.03	0.55–46.47	0.154
**Driver education**	**Driver education**	Town	**Driver education**					
			No formal education	64.3% (18/28)	35.7% (10/28)	1.00		
			Primary education	79.4% (100/126)	20.6% (26/126)	2.25	1–5.55	0.078
			Early secondary	74.8% (80/107)	25.2% (27/107)	2.14	1–5.4	0.106
			Late secondary	63.4% (64/101)	36.6% (37/101)	1.39	0.5–3.52	0.483
			Tertiary education	80.0% (4/5)	20.0% (1/5)	3.03	0.33–31.95	0.357
**Hitching**	**Traction through point of draught (POD); Functional breeching**	Equipment quality, Training source, Work type	**Traction through point of draught (POD)**					
			Through POD	64.4% (85/132)	35.6% (47/132)	1.00		
			Not through POD	77.1% (182/236)	22.9% (54/236)	1.5	0.86–2.62	0.154
**Equipment quality**	**Presence of traces; neck-strap; swingle tree; breeching; equipment maintenance; cost of cart**	Equipment source, Training source, Work type	**Cost of cart** *(per 100 ETB unit)*	*n/a*	*n/a*	Coef. –0.003	–0.006 – –0.001	0.182
**Source of equipment**	**Source of equipment**	Town, Training source, Work type	**Source of equipment**					
			Partly purchased & home-made	51.6% (16/31)	48.4% (15/31)	1.00		
			Purchased	74.2% (230/310)	25.8% (80/310)	3.36	1.36–8.29	0.009
			Inherited	91.7% (11/12)	8.3% (1/12)	12.15	1.34–109.95	0.026
			Donated	69.2% (9/13)	30.8% (4/13)	2.23	0.47–10.54	0.311
**Training source**	**Source of training on hitching and assembly; Source of further information about equipment**	Driver education, Town, Work type	**Source of training on hitching and assembly**					
			Other drivers	75.3% (67/89)	24.7% (22/89)	1.00		
			Intuition & observation	71.8% (183/255)	28.2% (72/255)	0.99	0.52–1.86	0.965
			Harness maker	70.8% (17/24)	29.2% (7/24)	1.03	0.37–2.88	0.958
**Welfare standard**	**Wound history; Measures to prevent wounds; Body condition score (BCS)**	Equipment quality, Hitching, Species	**Wound history**					
			No history of wounds	34.9% (30/86)	65.1% (56/86)	1.00		
			History of wounds	84% (237/282)	16% (45/282)	9.71	5.47–17.2	< 0.001

Assessing total effects, only town, equipment source and prior history of wounds were significant predictors of equipment-related wounds after minimal sufficient adjustments (Table 3; Figure 2). Animals from Shashamene were more likely to have wounds than those from Fiche (OR 2.56; 95% CI 1.40–4.63) or Bishoftu (OR 2.22; 95% CI 1.20–4.07; *P* = 0.006), purchased equipment was more likely to be associated with wounds than partly purchased and partly home-made equipment (OR 3.36; 1.36–8.29; *P* = 0.009), and animals with a history of wounds were more likely to have equipment-related wounds than those with no previous history (OR 9.71; 95% CI 5.47–17.20; *P* < 0.001) (Table 3).

#### Presence of individual equipment-related wound types

Significant associations between population and equipment characteristics and the presence of specific types of equipment-related wounds are presented in Table 4. Specific equipment characteristics were associated with certain wound types: for example, narrow saddle pressure points (OR 2.83; 95% CI 1.62–4.95; *P* < 0.001) and insufficient (OR 2.55; 95% CI 1.41–4.55) or excessive (OR 3.24; 95% CI 1.35–7.78; *P* = 0.004) saddle padding were associated with the presence of saddle wounds (Table 4). Poor bit fit (OR 0.24; 95% CI 0.08–0.68; *P* = 0.007) and condition of the bit (OR 0.18; 95% CI 0.04–0.76; *P* = 0.02) were associated with the presence of bit wounds.

**Table 4.** Significant predictors of specific types of equipment-related wounds through univariable logistic regression analysis in a study investigating the prevalence and factors associated with equipment-related wounds in Ethiopian working equids in 2022. Comparison of species was done between horses and donkeys only. Due to high collinearity, associations with town are not represented in this table

**Significant predictors of** **equipment-related wounds**	Wounds	No Wounds	Odds Ratio (OR)	95% Confidence Interval (OR)	Significance value (*P*)
**Significant predictor of saddle wounds**					
Saddle pressure points					
Wide pressure points	17.7% (52/294)	82.3% (242/294)	1.00		
Narrow pressure points	37.8% (28/74)	62.2% (46/74)	2.83	1.62–4.95	< 0.001
Thickness of saddle padding					
Adequate padding thickness	12.4% (17/137)	87.6% (120/137)	1.00		
Insufficient padding thickness	26.5% (52/196)	73.5% (144/196)	2.55	1.41–4.55	0.002
Excessive padding thickness	31.4% (11/35)	68.6% (24/35)	3.24	1.35–7.78	0.009
**Significant predictor of collar wounds**					
Species					
Horses	27% (66/244)	73% (178/244)	1.00		
Donkeys	15.6% (19/122)	84.4% (103/122)	0.50	0.29–0.88	0.015
Presence of traces					
Traces present	26.8% (66/246)	73.2% (180/246)	1.00		
Traces not present	16.4% (20/122)	83.6% (102/122)	0.54	0.31–0.93	0.027
**Significant predictor of girth wounds**					
Species					
Horses	50% (122/244)	50% (122/244)	1.00		
Donkeys	30.3% (37/122)	69.7% (85/122)	0.44	0.28–1.00	< 0.001
Breathability of girth materials *(when girth present)*					
Non-breathable materials	35.2% (38/108)	64.8% (70/108)	1.00		
Breathable materials	47.6% (118/248)	52.4% (130/248)	1.67	1.05–2.67	0.031
Work Type					
Taxi work	49.4% (115/233)	50.6% (118/233)	1.00		
Transport of goods	29.2% (31/106)	70.8% (75/106)	0.43	0.26–0.70	< 0.001
**Significant predictor of bit wounds**					
Species					
Horses	3.7% (9/244)	96.3% (235/244)	1.00		
Donkeys	31.1% (38/122)	68.9% (84/122)	11.80	5.48–25	< 0.001
Bit condition					
Poor condition	15% (45/301)	85% (256/301)	1.00		
Good condition	3.1% (2/65)	96.9% (63/65)	0.18	0.04–0.76	0.020
Bit size/fit					
Doesn’t fit the animal	15.8% (43/272)	84.2% (229/272)	1.00		
Fits the animal	4.3% (4/94)	95.7% (90/94)	0.24	0.08–0.68	0.007
Main work					
Taxi work	3.4% (8/233)	96.6% (225/233)	1.00		
Transport of goods	31.1% (33/106)	68.9% (73/106)	12.71	5.62–28.76	< 0.001
Mixed work	21.1% (4/19)	78.9% (15/19)	7.50	2.03–27.77	0.003
**Significant predictor of blinker wounds**					
Species					
Horses	1.2% (3/244)	98.8% (241/244)	1.00		
Donkeys	7.4% (9/122)	92.6% (113/122)	6.4	1.7–24	0.006
Main work					
Taxi work	1.3% (3/233)	98.7% (230/233)	1.00		
Transport of goods	8.5% (9/106)	91.5% (97/106)	6.42	1.83–22.45	0.004
**Significant predictor of limb wounds attributable to equipment**					
Species					
Horses	2% (5/244)	98% (239/244)	1.00		
Donkeys	15.6% (19/122)	84.4% (103/122)	8.8	3.21–24	< 0.001
Breeching breathability *(when breeching present)*					
Non-breathable materials	13.3% (14/105)	86.7% (91/105)	1.00		
Breathable materials	4.1% (7/171)	95.9% (164/171)	0.28	0.11–0.71	0.008
Main work					
Taxi work	1.7% (4/233)	98.3% (229/233)	1.00		
Transport of goods	17.9% (19/106)	82.1% (87/106)	11.37	3.95–32.73	< 0.001
**Significant predictor of shaft wounds**					
Species					
Horses	0.8% (2/244)	99.2% (242/244)	1.00		
Donkeys	59% (72/122)	41% (50/122)	174.24	41.38–733.64	< 0.001
Main work					
Taxi work	0.9% (2/233)	99.1% (231/233)	1.00		
Transport of goods	56.6% (60/106)	43.4% (46/106)	120.48	32.58–445.58	< 0.001
Mixed work	57.9% (11/19)	42.1% (8/19)	125.28	27–581.2	< 0.001
Transport of water	18.2% (2/11)	81.8% (9/11)	24.368	3.74–158.98	< 0.001

## Discussion

This study investigated the prevalence and factors associated with equipment-related wounds in working equids in three Ethiopian locations. A high overall prevalence of equipment-related wounds was found affecting 7 out of 10 working equids, thus representing a serious welfare concern for these animals. This study suggests that the likelihood of equipment-related wounds is influenced by species, town, work-type and equipment source, and that animals with a low welfare standard are more likely to have equipment-related wounds. Additionally, specific equipment characteristics and practices influenced the likelihood of having equipment-related wounds.

More than two-thirds of the equids in this study had at least one type of equipment-related wound. Although reporting of the causes and classification of lesions are not generally standardised, and the aetiology of wounds is multifactorial and could be influenced by several factors external to the equipment, such as age, work-type or low body condition (Pritchard *et al.* 2005; Burn *et al.* 2010; Sánchez-Casanova *et al.* 2014); this is among the highest prevalence reported for the Oromia region (Usman *et al.* 2015). Donkeys had a higher prevalence of equipment-related wounds than horses affecting 8 out of 10 donkeys, which is consistent with other studies (Demelash Biffa 2006; Usman *et al.* 2015). Other authors suggest a higher wound prevalence in horses (Kompi *et al.* 2023) or no significant species differences (Fikru *et al.* 2015), which may be due to different study regions and designs, or differing risk factors and environmental contexts. However, donkeys have frequently been reported to have a lower welfare standard than horses (Sells *et al.* 2010; Haddy *et al.* 2021) and a lower perceived value (Fernando & Starkey 2004; Geiger *et al.* 2020). Additionally, previous history of wounds was strongly associated with equipment-related wounds, suggesting poor welfare sustains further welfare compromise. This could be explained by a failure to adequately address the root causes of the problem, such as equipment design, work or husbandry practices. Similarly, the association between town and wounds may reflect regional variations in the standard of welfare, species, work type or quality of equipment among other factors. As such, Shashamene may have a higher prevalence of wounds because donkeys are more common than horses in that town, but potentially also due to other environmental, population or contextual characteristics within the region. Town, work-type, history of wounds and species were not independent of each other, and thus it could not be determined whether it was the individual factor, or a combination of them that increased the risk of wounds. Nonetheless, this suggests the issue varies according to location and population of animals and targeted advice and solutions are therefore required.

The absence of certain equipment components was associated with a greater likelihood of having equipment-related wounds, which can be expected given the role and described function of these components within a harnessing system. For example, the presence of a neck strap, traces and swingle tree within a breast collar harness system is designed to enable efficient traction through the collar, maintain it in an appropriate position and reduce its friction against the animal’s shoulders during movement (Pearson *et al.* 2003; Oudman 2004). Therefore, the absence of these components would not only decrease efficiency of traction but also reasonably increase the likelihood of friction wounds in the region of the collar as observed in this study. When considering specific types of equipment-related wounds, lesions in the region of the girth were the overall most common type of wound, followed by collar and saddle wounds. However, this differed between species and while girth, collar and saddle wounds were most prevalent in horses, which is similar to reports from other countries (Pritchard *et al.* 2005; Teferi *et al.* 2020), wounds in the region of the shafts and bit were the most prevalent in donkeys. This suggests equipment characteristics vary between horses and donkeys with equipment used for donkeys generally being of a lower standard (Merridale-Punter *et al.* 2024).

Several equipment characteristics were also associated with specific types of equipment-related wounds. For example, saddle sores were associated with saddles that had narrow pressure points or excessive or insufficient levels of padding, and bit wounds were associated with a poor bit fit and condition. Some associations were less intuitive and potentially contrary to recommendations (Pearson *et al.* 2003; Heleski *et al.* 2009; Garrett 2017): for example, girths made from synthetic materials were less associated with wounds than those made from fabrics or other natural, breathable materials. This is a similar finding to what has been previously found for strap lesions in working donkeys (Burn *et al.* 2008), and could reflect the fact that more porous materials can easily absorb sweat and dirt which, if not adequately cleaned and maintained, may make them stiffer, dirtier and more abrasive. Similarly, an excessive level of saddle padding would increase under-saddle heat, pressure and sweating, increasing friction and the likelihood of wounds (Garrett 2017), particularly if the girth is loose. Draught dynamics are complex, and equipment components cannot be viewed in isolation but rather considered as part of a dynamic interacting unit which is also influenced by factors such as work practices, fitting, loading and weight distribution. As such, equipment practices such as maintenance must also be considered in the context of wound prevention. Nonetheless, these findings provide guidance for specific adjustments in the design or use of individual components. Prioritising targeted improvement of existing equipment, rather than replacing entire harness designs, could help increase user uptake of preventive strategies in a more cost-effective way (Chanie *et al.* 2012).

Equipment source was a significant predictor of equipment-related wounds, with purchased and particularly inherited equipment being more associated with the presence of wounds than partially home-made equipment. As suggested in our previous study, equipment may be being purchased from sources where the design or materials are not fit for purpose, potentially due to a lack of technical knowledge or capacity among equipment makers (Merridale-Punter *et al.* 2024). As the cost of the harness was not associated with the presence of wounds, this could also suggest that simple, home-made or inexpensive harness components may be efficient and fit for purpose if designed appropriately, as reported by other authors for different full-collar designs (Rodrigues *et al.* 2021). This becomes particularly relevant in the context of the economic pressures faced by most working equid users (Geiger *et al.* 2020) and given that cost is often the main motivator and barrier to obtaining the ideal work equipment (Merridale-Punter *et al.* 2024). Partial home-made production or adaptations may also reflect a higher standard of care for the equid, and may allow for equipment adjustments that provide a better fit to the individual animal or species, improving its comfort and efficiency where a range of equipment models and sizes may not be commercially available or affordable (Rodrigues *et al.* 2021; Merridale-Punter *et al.* 2024). Conversely, carts that cost less were associated with presence of equipment-related wounds. Carts are more complex items to produce at home, and it can be reasonably assumed that most carts are purchased from manufacturers. This suggests there may be price-related differences in cart design and quality as well as knowledge gaps among manufacturers, and highlights the importance of considering the vehicle itself and the way in which it is designed, hitched, loaded and used as an extension of the work equipment (Pearson *et al.* 2003; Oudman 2004; Garrett 2017). As such, understanding the principles of harnessing and equipment design and function is not only relevant for cart and harness makers but also to working equid users and could be incorporated in community and professional training initiatives aimed at improving equipment standards. It should be noted that most participants purchased their equipment with no differentiation between individual producers or markets considered in this study, and equipment source was not independent of town and other predictors of wounds. There may therefore be region-specific variations in the standard of equipment sources which requires further investigation.

In this study, demographic characteristics of cart drivers were not associated with equipment-related wounds, unlike other reports from Ethiopia where lower driver education was associated with greater prevalence of wounds and other indicators of poor welfare (Molla *et al.* 2017; Teferi *et al.* 2020). Similarly, income comfort level was not associated with equipment-related wounds (and, in fact, exclusively purchased equipment was associated with wounds), which supports findings from other reports that suggest working equid welfare does not necessarily correlate with the owner’s socioeconomic status and level of education (Bazezew *et al.* 2014; Lanas *et al.* 2018). Conversely, the prevalence of equipment-related wounds was associated with hitching and harnessing practices, where not fitting the breast collar over the animal’s point of draught (POD) was almost twice as likely to be associated with wounds compared to a collar positioned over the POD. This is in line with existing harnessing recommendations, increasing comfort and efficiency of traction as well as decreasing the likelihood of injury (Pearson *et al.* 2003; Oudman 2004; Davis 2014). Once more, this also suggests that in addition to the equipment itself, the practices and ways in which the equipment is used must also be considered part of prevention strategies. As such, there is an opportunity for education on equipment design and practices, where human behaviour change could lead to a decrease in equipment-related wounds regardless of demographic or socioeconomic constraints.

There are several limitations to this study. Equipment-related wounds were recorded based on their location in relation to the equipment, but we did not have sufficient information to confirm their cause. Further longitudinal studies would be required to confirm the causal factors of equipment-related wounds. Additionally, the effects of equipment components on wounds and other welfare indicators cannot be looked at in isolation as they are part of an interconnected animal-harness-cart apparatus with complex interacting traction dynamics, which would require investigation in motion and under controlled conditions of work intensity, environment, load distribution, among other factors. Nonetheless, important insights into specific equipment characteristics associated with wounds were found in a non-invasive observational manner, identifying key areas for improvement and further investigation. We classified wounds in accordance with the SEBWAT tool definition of lesions (Sommerville *et al.* 2018), ranging from superficial or healed skin lesions to deep open wounds, which may influence prevalence results in relation to other studies using alternative classifications. However, descriptive results of wound size and severity are provided for transparency, and this allows for result comparison with other literature using the same standardised assessment tool. The temperament and behaviour of equids may differ between castrated and entire males and should be considered in future research as it may affect the prevalence of wounds. Some equipment classifications may be influenced by the point within the workday when the animal was surveyed among other factors. However, limiting all data collection to two trained researchers reduced misclassification bias. Finally, regional differences may mean these findings are not applicable to other sectors of the population, and the multicollinearity obtained between town, species, work type and other predictor variables prevents accurate determination of the influence of each individual predictor on the likelihood of equipment-related wounds. Nonetheless, this work offers further characterisation of potentially problematic equipment attributes than previous literature focusing on overall adequacy or inadequacy of equipment; and highlights equipment-related wounds as a complex and multifactorial welfare issue requiring context-specific solutions.

### Animal welfare implications

Equipment-related wounds are highly prevalent in Ethiopian working equids thereby posing an important welfare concern. Although a better understanding of work-related risk factors for welfare is still needed; species, work-type and regional differences in both the prevalence and the type of equipment-related wounds suggest that tailored preventive strategies and equipment adaptations are required. Driver demographics and economic comfort-level did not influence the presence of equipment-related wounds, and several equipment attributes associated with wounds could be tangibly modifiable without significant cost implications. Equipment source, harnessing and hitching practices also affected the likelihood of having equipment-related wounds, highlighting the importance of including human behaviour change as part of wound prevention strategies. A better understanding of equipment function, assembly and use among different stakeholders is therefore needed to safeguard the welfare of working equids, and training and education efforts should be targeted not only at cart drivers for improved harnessing, hitching and equipment care practices, but also at harness and cart makers for improved equipment design.

## Supporting information

Merridale-Punter et al. supplementary material 1 Merridale-Punter et al. supplementary material

Merridale-Punter et al. supplementary material 2 Merridale-Punter et al. supplementary material

Merridale-Punter et al. supplementary material 3 Merridale-Punter et al. supplementary material

Merridale-Punter et al. supplementary material 4 Merridale-Punter et al. supplementary material

## Supplementary material

The supplementary material for this article can be found at http://doi.org/10.1017/awf.2024.52.
