# Supplementary material for: Equipment-related wounds and associated risk factors in working equids of the Oromia national regional state in Ethiopia
Source: Anim Welf. 2024 Oct 31;33:e42. doi: 10.1017/awf.2024.52 (PMC11589070; doi:10.1017/awf.2024.52)
Supplement: Merridale-Punter et al. supplementary material 2 — Merridale-Punter et al. supplementary material [file S0962728624000526sup002.pdf]

## Supplement 2 – Variables tested as predictors of equipment-related wounds

| Predictors Investigated | | Outcome |
|---|---|---|
| Town | Driver thinks of load distribution | Overall presence of equipment-related wounds |
| Species | Driver would like to change something about equipment | |
| BCS | Belief of harness influencing ability to work | |
| Main type of work | Belief of cart influencing ability to work | |
| Number of days worked per week | Belief whether equipment is comfortable for the animal | |
| Average number of hours worked per day | Belief whether equipment is efficient | |
| Average number of breaks per day | Belief whether equipment is assembled correctly | |
| Average duration of breaks per day | Removing harness during breaks | |
| Cleanliness of harness | History of wounds | |
| Cart axel balance | Measures to prevent wounds | |
| Shafts passing through the centre of gravity | Being responsible for assembling and hitching | |
| Traction through point of draught | Source of training on hitching and assembly | |
| Driver age | Source of information about equipment | |
| Driver number of dependents | Presence of girth | |
| Percentage of income derived from equid | Presence of belly band | |
| Driver Education | Presence of crupper | |
| Driver gender | Presence of back-band | |
| Ownership of equid | Presence of traces | |
| Income comfort level | Presence of neck strap | |
| Income percentage derived from equid | Presence of swingle | |
| Years of experience driving | Presence of breeching | |
| Years of experience with equids | Saddle positioned adequately | |
| Level of enjoyment of the profession | Presence of a functional swingle tree*1 | |
| Source of equipment | Presence of a functional saddle*2 | |
| Source of training as a driver | Presence of functional traction*3 | |
| Cost of harness | Presence of functional breeching*4 | |
| Cost of cart | | |
| Priority when choosing equipment | **1 Swingle tree is present and moves freely. Traces attach to swingle tree.* | |
| Equipment receiving maintenance | **2 Adequately positioned, wide pressure points, has gullet, has padding, secured tightly to the animal.* | |
| Person responsible for maintenance | **3 Adequate position over the point of draught, freely moving traces that attach to a functional swingle tree* | |
| Frequency of harness maintenance | **4 Presence of breeching and correctly assembled as either true or false breeching* | |
| Frequency of cart maintenance | | |

| Predictors Investigated | | Outcome |
|---|---|---|
| Town | Thickness of saddle padding | Saddle Wounds |
| Species | Saddle shape (presence of gullet) | |
| Breathability of saddle padding | Shafts passing through the centre of gravity | |
| Saddle fit | Main type of work | |
| Saddle position | Cart axel balance | |
| Saddle pressure | Functional saddle | |

| Predictors Investigated | | Outcome |
|---|---|---|
| Town<br>Species<br>Presence of breast collar padding<br>Breathability of breast collar<br>Breathability of breast collar padding<br>Breathability of breast collar contact surface<br>Position of the breast collar<br>Breast collar passing through point of draught<br>Presence of a functional swingle tree | Breast collar being adjustable<br>Breast collar width<br>Presence of traces<br>Free movement of traces (when traces present)<br>Adequacy (thickness) of breast collar padding<br>Cart axel balance<br>Main type of work<br>Functional traction | Breast collar wounds |

| Predictors Investigated | | Outcome |
|---|---|---|
| Town<br>Species<br>Breathability of girth materials | Main type of work<br>Functional girth | Girth Wounds |

| Predictors Investigated | | Outcome |
|---|---|---|
| Town<br>Species<br>Bit condition | Bit size/fit<br>Free movement of reins<br>Main type of work | Bit wounds |

| Predictors Investigated | | Outcome |
|---|---|---|
| Town<br>Species<br>Blinker position | Blinker shape<br>Main type of work | Blinker wounds |

| Predictors Investigated | | Outcome |
|---|---|---|
| Town<br>Species<br>Breeching presence<br>Breeching type | Breeching assembly<br>Breeching breathability<br>Main type of work<br>Functional breeching | Limb wounds attributable to equipment |

| Predictors Investigated | | Outcome |
|---|---|---|
| Town<br>Species<br>Crupper presence<br>Crupper breathability | Breeching presence<br>Breeching type<br>Breeching assembly Main type of work<br>Functional breeching | Tail wounds attributable to equipment |

| Predictors Investigated | | Outcome |
|---|---|---|
| Town<br>Species<br>Shafts width | Shafts passing through centre of gravity<br>Cart axel balance<br>Main type of work | Shaft Wounds |
