# Supplementary material for: Equipment-related wounds and associated risk factors in working equids of the Oromia national regional state in Ethiopia
Source: Anim Welf. 2024 Oct 31;33:e42. doi: 10.1017/awf.2024.52 (PMC11589070; doi:10.1017/awf.2024.52)
Supplement: Merridale-Punter et al. supplementary material 3 — Merridale-Punter et al. supplementary material [file S0962728624000526sup003.pdf]

Supplement 3 – Welfare assessment results

Supplement 3, Table 1 – Welfare assessment [1] results in a cross-sectional study investigating the prevalence and factors associated with equipment-related wounds in Ethiopian working equids. Clustered by equid species.

Welfare Indicator	Horses		Donkeys		Mules		Overall Equids	
**General attitude [1]**								
Positive	72.5%	177/244	77.9%	95/122	100%	3/3	74.5%	275/369
Non-reactive	0.4%	1/244	4.9%	6/122	0%	0/3	1.9%	7/369
Negative	27.0%	66/244	17.2%	21/122	0%	0/3	23.6%	87/369
**Response to spinal contact [1]**								
No reaction	3.3%	8/244	4.9%	6/122	66.7%	2/3	4.3%	16/369
Reaction	96.7%	236/244	95.1%	116/122	33.3%	1/3	95.7%	353/369
**Body condition score (BCS) [1]**								
Mean BCS ±SD	2.00	±0.68	1.89	±0.38	1.67	±0.58	1.96	±0.60
**Limb score (forelimbs) [1]**								
Mean score ±SD	1.16	±0.45	1.02	±0.24	1.67	±1.15	1.12	±0.41
**Limb score (hindlimbs) [1]**								
Mean score ±SD	0.15	±0.44	0.07	±0.33	0.67	±1.15	0.13	±0.42
**Hoof score (forelimbs) [1]**								
Mean score ±SD	1.61	±0.89	1.02	±0.18	1.67	±1.15	1.41	±0.79
**Hoof score (hindlimbs) [1]**								
Mean score ±SD	1.72	±0.87	1.02	±0.20	1.67	±1.15	1.49	±0.79
**Gait assessment [1]**								
Not compromised	67.2%	164/244	85.2%	104/122	100%	3/3	72.6%	271/369
Moderately compromised	27.9%	68/244	13.9%	17/122	0%	0/3	23.0%	85/369
Highly compromised	4.9%	12/244	0.8%	1/122	0%	0/3	3.5%	13/369
Unable to bear weight	0%	0/244	0%	0/122	0%	0/3	0%	0/369
**Prevalence of Wounds**								
Equipment-related wounds	67.6%	165/244	82.0%	100/122	100%	3/3	72.6%	268/369
Any type of wounds	71.3%	174/244	84.4%	103/122	100%	3/3	75.9%	280/369

Supplement 3, Table 2 - Welfare assessment [1] results in a cross-sectional study investigating the prevalence and factors associated with equipment-related wounds in Ethiopian working equids. Clustered by study location.

Welfare Indicator	Bishoftu		Fiche		Shashamene	
**General attitude [1]**						
Positive	71.3%	87/122	74.0%	91/123	78.2%	97/124
Non-reactive	0.8%	1/122	0%	0/123	4.8%	6/124
Negative	27.9%	34/122	26.0%	32/123	16.9%	21/124
**Response to spinal contact [1]**						
No reaction	0%	0/122	6.5%	8/123	4.8%	6/124
Reaction	100%	122/122	93.5%	115/123	95.2%	118/124
**Body condition score (BCS) [1]**						
Mean BCS ±SD	1.99	±0.65	2.02	±0.72	1.88	±0.37
**Limb score (forelimbs) [1]**						
Mean score ±SD	1.18	±0.53	1.12	±0.35	1.05	±0.31
**Limb score (hindlimbs) [1]**						
Mean score ±SD	0.16	±0.46	0.15	±0.42	0.08	±0.37
**Hoof score (forelimbs) [1]**						
Mean score ±SD	1.05	±0.38	2.16	±0.91	1.03	±0.25
**Hoof score (hindlimbs) [1]**						
Mean score ±SD	1.16	±0.43	2.27	±0.85	1.04	±0.27
**Gait assessment [1]**						
Not compromised	72.1%	88/122	63.4%	78/123	84.7%	105/124
Moderately compromised	21.3%	26/122	33.3%	41/123	14.5%	18/124
Highly compromised	6.6%	8/122	3.3%	4/123	0.8%	1/124
Unable to bear weight	0%	0/122	0%	0/123	0%	0/124
**Prevalence of Wounds**						
Harness-related wounds	68.9%	84/122	65.9%	81/123	83.1%	103/124
Any type of wounds	75.4%	92/122	67.5%	83/123	84.7%	105/124

[1] Sommerville R, Brown AF, Upjohn M. A standardised equine-based welfare assessment tool used for six years in low and middle income countries. PLoS One. 2018;13(2):e0192354.

Supplement 3, Table 3 - Number, size and severity of equipment-related wounds in a cross-sectional study investigating the prevalence and factors associated with equipment-related wounds in Ethiopian working equids. Only animals with wounds are included in this description. The median and interquartile range (IQR) are presented.

	**Horses**						**Donkeys**						**Overall Equids**					
	**Number of wounds**		**Wound Severity**		**Wound Size**		**Number of wounds**		**Wound Severity**		**Wound Size**		**Number of wounds**		**Wound Severity**		**Wound Size**	
**Wound type**	**Median**	**IQR**	**Median**	**IQR**	**Median**	**IQR**	**Median**	**IQR**	**Median**	**IQR**	**Median**	**IQR**	**Median**	**IQR**	**Median**	**IQR**	**Median**	**IQR**
BIT wounds	1	1-2	2	1-2	1	1-1	2	1-2	2	1.3-2	1	1-2	2	1-2	2	1-2	1	1-1.5
BLINKER wounds	1	1-1.5	2	2-2	1	1-1.5	1	1-2	1	1-2	1	1	1	1-2	1.5	1-2	1	1
SADDLE wounds	1	1-2	1	1-2	2	1-2	2	2-2	1	1-2	2	1-3	1.5	1-2	1	1-2	2	1-2
COLLAR wounds	1	1-2	1	1-2	2	1-2	2	2-2	1	1	2	2-3	2	1-2	1	1-2	2	1-2
GIRTH wounds	2	2	2	1-2	2	1-2	2	1-2	1	1-2	2	2	2	1-2	1	1-2	2	1-2
SHAFT wounds	1	1	1.5	1-2	1	1	2	2	1	1-2	2	2	2	2	1	1-2	2	2
LIMB wounds attributable to equipment	2	1-2	2	2	2	1-2	2	1.5-2	1	1	2	2	2	1-2	1	1-2	2	2
TAIL wounds attributable to equipment	1	1	2	2	2	2	1	1	2	2	2	2	1	1	2	2	2	2
