# Supplementary material for: Equipment-related wounds and associated risk factors in working equids of the Oromia national regional state in Ethiopia
Source: Anim Welf. 2024 Oct 31;33:e42. doi: 10.1017/awf.2024.52 (PMC11589070; doi:10.1017/awf.2024.52)
Supplement: Merridale-Punter et al. supplementary material 4 — Merridale-Punter et al. supplementary material [file S0962728624000526sup004.pdf]

Supplement 4 – Description of harness components and characteristics and structured interviews

Supplement 4, Table 1 – Description of the proportion and frequency of harness components and characteristics in a cross-sectional study investigating work-equipment and practices in working equids of three Ethiopian locations.

Equipment Components Present<br>Equipment characteristics	Equipment-related wounds		No equipment-related wounds		All equids	
	Frequency	%	Frequency	%	Frequency	%
Bit	267/366	73%	99/366	27%	366/368	99.5%
Good condition	44/65	67.7%	21/65	32.3%	65/366	17.8%
Correct size/fit	69/94	73.4%	25/94	26.6%	94/366	25.6%
Blinkers	233/323	72.1%	90/323	27.9%	323/368	87.8%
Dished shape	75/102	73.5%	27/102	26.5%	102/323	31.6%
Not touching the eyelashes	81/115	70.4%	34/115	29.6%	115/323	35.6%
Reins	267/366	73%	99/366	27%	366/368	99.5%
Freely moving	265/364	72.8%	99/364	27.2%	364/366	99.5%
Breast collar	267/368	72.6%	101/368	27.4%	368/368	100%
Collar padding	108/145	74.5%	37/145	25.5%	145/368	39.4%
Positioned through point of draught	85/132	64.4%	47/132	35.6%	132/368	35.9%
Easily adjustable	8/10	80%	2/10	20%	10/368	2.7%
Made from breathable contact materials	188/259	72.6%	71/259	27.4%	259/368	70.4%
Neck strap	137/211	64.9%	74/211	35.1%	211/368	57.3%
Easily adjustable	12/16	75%	4/16	25%	16/211	7.6%
Made from breathable contact materials	65/101	64.4%	36/101	35.6%	101/211	47.9%
Traces	166/246	67.5%	80/246	32.5%	246/368	68.8%
Freely moving	90/134	67.2%	44/134	32.8%	134/246	54.5%
Swingle tree	101/151	66.9%	50/151	33.1%	151/368	41%
Freely moving	52/72	72.2%	20/72	27.8%	72/151	47.7%
With traces attaching	100/150	66.7%	50/150	33.3%	150/151	99.3%
Functional swingle tree*[1]	52/72	72.2%	20/72	27.8%	72/368	19.6%
Functional traction*[2]	13/17	76.5%	4/17	23.5%	17/368	4.6%
Saddle	267/368	72.6%	101/368	27.4%	368/368	100%
Includes a gullet	134/203	66%	69/203	34%	203/368	55.2%
Positioned at base of the withers	181/246	73.6%	65/246	26.4%	246/368	66.8%
Wide pressure points	211/294	71.8%	83/294	28.2%	294/368	79.9%
Secured tightly in place (girth not loose)	126/173	72.8%	47/173	27.2%	173/368	47%
Saddle padding	267/368	72.6%	101/368	27.4%	368/368	100%
Padding made from breathable contact materials	65/94	69.1%	29/94	30.9%	94/368	25.5%
Functional saddle*[3]	21/33	63.6%	12/33	36.4%	33/368	9.0%
Back band	61/86	70.9%	25/86	29.1%	86/368	23.4%
Belly band	43/63	68.3%	20/63	31.7%	63/368	17.1%
Girth	258/356	72.5%	98/356	27.5%	356/368	96.7%
Easily adjustable	250/347	72%	97/347	28%	347/356	97.5%
Made from breathable contact materials	166/248	66.9%	82/248	33.1%	248/356	69/7%
Crupper	152/214	71%	62/214	29%	214/368	58.2%
Made from breathable contact materials	78/117	66.7%	39/117	33.3%	117/214	54.7%
Tugs	261/361	72.2%	100/361	27.7%	361/368	98.1%
Breeching	195/277	70.3%	82/277	29.6%	277/368	75.3%
True breeching	189/269	70.3%	80/269	29.7%	269/277	97.1%
False breeching	6/8	75%	2/8	25%	8/277	2.9%
Correct breeching assembly	39/60	65%	21/60	35%	60/277	21.7%
Made from breathable contact materials	115/171	67.3%	56/171	32.7%	171/277	61.7%
Functional breeching*[4]	39/60	65%	21/60	35%	60/368	16.3%
Shafts passing through the centre of gravity	91/122	74.6%	31/122	25%	122/368	33.2%
Harness is clean	9/15	60%	6/15	40%	15/368	4.1%
Balanced cart axel	256/353	72.5%	97/353	27.5%	353/368	95.9%

**1 Swingle tree is present and moves freely. Traces attach to swingle tree.*

**2 Adequate position over the point of draught, freely moving traces that attach to a functional swingle tree*

**3 Adequately positioned, wide pressure points, has gullet, has padding, secured tightly to the animal.*

**4 Presence of breeching and correctly assembled as either true or false breeching*

Supplement 4, Table 2 – Description of the proportion and frequency of structured interview questions to working equid cart-drivers in a cross-sectional study investigating work-equipment and practices in working equids of three Ethiopian locations.

**Structured Interview Questions**	**Equipment-related wounds**		**No equipment-related wounds**		**All equids**	
	**Median**	**Range**	**Median**	**Range**	**Median**	**Range**
Driver age (years)	29	24 - 70	28	18 - 68	28	24 - 70
Experience with equids (years)	4	1 - 32	4	1 - 22	4	1 - 32
Cost of harness (Ethiopian Birr)	4,000	500 – 25,000	4,000	1,000 – 20,000	4,000	500 - 25,000
Cost of cart (Ethiopian Birr)	17,000	1,200 – 45,000	20,000	1,500 – 50,000	18,000	1,200 - 50,000
	**Frequency**	**%**	**Frequency**	**%**	**Frequency**	**%**
Driver gender						
Female	2/4	50%	2/4	50%	4/368	1.1%
Male	265/364	72.8%	99/364	27.2%	364/368	98.9%
Ownership of equid						
Owner and driver	248/343	72.3%	95/343	27.7%	343/368	93.2%
Driver only	19/25	76%	6/25	24%	25/368	6.8%
Driver Education						
No formal education	18/28	64.3%	10/28	35.7%	28/368	7.6%
Primary education	100/126	79.4%	26/126	20.6%	126/368	34.2%
Early secondary	80/107	74.8%	27/107	25.2%	107/368	29.1%
Late secondary	64/101	63.4%	37/101	36.6%	101/368	27.4%
Tertiary education	4/5	80%	1/5	20%	5/368	1.4%
Prefer not to say	1/1	100%	0/1	0%	1/368	0.3%
Economic comfort level						
Finding it difficult	17/22	77.3%	5/22	22.7%	22/368	6.0%
Just managing	183/250	73.2%	67/250	26.8%	250/368	67.9%
Comfortable	67/96	69.8%	29/96	30.2%	96/368	26.1%
Very Comfortable	*n/a*	*n/a*	*n/a*	*n/a*	0/368	0%
Prospering	*n/a*	*n/a*	*n/a*	*n/a*	0/368	0%
Source of training about harness assembly and hitching						
Observation and intuition	183/344	53.2%	72/344	20.9%	344/468	73.5%
Other drivers	67/100	67%	22/100	22%	100/468	21.3%
Harness maker	17/24	70.8%	7/24	29.2%	24/468	5.1%
Equipment source						
Purchased	230/310	74.2%	80/310	25.8%	310/366	84.7%
Partly Purchased & Home-made	16/31	51.6%	15/31	48.4%	31/366	8.5%
Donated	9/13	69.2%	4/13	30.8%	13/366	3.6%
Inherited	11/12	91.7%	1/12	8.3%	12/366	3.3%
Equipment receives routine maintenance	220/308	71.4%	88/308	28.6%	308/368	83.7%
Previous history of wounds	237/282	84%	45/282	16%	282/368	76.6%
Frequency of wound history:						
Rarely	82/112	73.2%	30/112	26.8%	112/368	30.4%
Occasionally	101/109	92.7%	8/109	7.3%	109/368	29.6%
Often	48/55	87.3%	7/55	12.7%	55/368	14.9%
Very Often	6/6	100%	0/6	0%	6/368	1.6%
Took measures to prevent wounds	85/106	80.2%	21/106	19.8%	106/368	28.8%
Wound Prevention measures:						
Seek veterinary care	36/45	80%	9/45	20%	45/368	12.2%
Wash or cover developing wounds	32/34	94.1%	2/34	5.9%	34/368	9.2%
Changing the equipment	9/19	47.4%	10/19	52.6%	19/368	5.2%
Traditional treatments	8/8	100%	0/8	0%	8/368	2.2%
